# An Evaluation of a Point-of-Care GEM Premier ChemSTAT Analyzer in an Emergency Department: Prevention of Contrast-Induced Nephropathy and Optimization of Patient Flow

**DOI:** 10.3390/jcm13237174

**Published:** 2024-11-26

**Authors:** Marina Brailova, Marie Audin, Julien Raconnat, Jean-Baptiste Bouillon-Minois, Jeannot Schmidt, Bruno Pereira, Damien Bouvier, Vincent Sapin

**Affiliations:** 1 Biochemistry and Molecular Genetic Department, CHU Clermont-Ferrand, F-63000 Clermont-Ferrand, France; mplatonov@chu-clermontferrand.fr (M.B.); maudinressot@chu-clermontferrand.fr (M.A.); dbouvier@chu-clermontferrand.fr (D.B.); 2 Adult Emergency Department, CHU Clermont-Ferrand, F-63000 Clermont-Ferrand, France; jraconnat@chu-clermontferrand.fr (J.R.); jjbbouillon-minois@chu-clermontferrand.fr (J.-B.B.-M.); jschmidt@chu-clermontferrand.fr (J.S.); 3 Biostatistics Unit (DRCI), CHU Clermont-Ferrand, F-63000 Clermont-Ferrand, France; bpereira@chu-clermontferrand.fr; 4 iGReD, School of Medicine, Santé Site, Clermont Auvergne University, F-63000 Clermont-Ferrand, France

**Keywords:** contrast media, creatinine, contrast-induced nephropathy, point-of-care, ChemSTAT, emergency

## Abstract

**Background:** Having a laboratory renal profile for medical imaging examinations requiring contrast media (CM) administration is strongly advised. Creatinine helps identify patients at risk of contrast-induced nephropathy (CIN). The GEM^®^ Premier™ ChemSTAT (Werfen) is a point-of-care (POC) analyzer with 12 emergency parameters, including a creatinine assay. This study aims to compare ChemSTAT with the central analytical solution of the University Hospital of Clermont-Ferrand and to evaluate the interest in using POC creatinine in the emergency department (ED) to optimize the flow of patients, especially when CM administration is necessary. **Methods:** More than 200 whole blood (WB) samples from the ED were evaluated on the ChemSTAT analyzer. As comparative methods, the plasma aliquots from the same samples were assayed on an Atellica^®^ CH (Siemens Healthineers). The clinical concordance was assessed according to the decision cut-offs of the French Society of Radiology for the risk of CIN. The availability times of biological results between ChemSTAT and the central laboratory were studied. **Results:** WB results from the ChemSTAT analyzer correlated well with those from the Atellica^®^ CH, except for tCO2 (the known bias between the Siemens and Cobas Roche methods for predicting ChemSTAT values). The results of the creatinine assay allow for identical medical decisions in comparison to the renal-risk cut-offs. The availability of the biological results was reduced by 50 min on average with ChemSTAT vs the central laboratory. Computed tomography (CT) was performed for 44.7% of patients, including the injection of the CM in 68% of cases. For these patients, the availability of creatinine results relative to imaging time is faster with the ChemSTAT by an average of 45.2 min. **Conclusions:** Great analytical and clinical correlations for creatinine assays allow for the safe identification of patients at risk of CIN, and improve patient flow in ED, especially for those requiring computed tomography with CM.

## 1. Introduction

Emergency department (ED) overcrowding has become an increasingly common problem around the world, along with other significant public health problems [1]. Therefore, the relevance of an early decision, which refers to the availability of reliable biological results, has become a challenge in overloaded emergency services. The point-of-care (POC) systems make it possible to obtain biological results more quickly and accelerate the overall management of patients. In addition to blood gas measurements, the availability of partial ionograms (sodium, potassium, chloride, and ionized calcium) and some substrates (glucose, creatinine, urea, and lactates) in POC make it possible to quickly diagnose and easily monitor acid-base and hydro-electrolytic disorders in whole blood (WB). The availability of creatinine allows for the quick assessment of the kidney function of patients in EDs, especially in order to decrease the risk of contrast-induced nephropathy (CIN), and also improves the organization of access to imaging procedures. The installation of such a POC system should, therefore, improve the flow of patients. It is, above all, necessary to ensure that the accuracy of point-of-care tests is equivalent to laboratory assays. Additionally, turnaround time improvements are not always clinically relevant, and the data in the literature are not all consistent [2]. In this study, we evaluated the GEM^®^ Premier™ ChemSTAT, a point-of-care system that measures multiple parameters (creatinine, urea, total carbon dioxide, electrolytes, glucose, hematocrit, pH, and pCO2) from a single sample of WB. These analytical performances have been confirmed by several studies [3,4], especially in our laboratory [5].

The present study focused on the comparison of methods for all analytes and the clinical concordance of the estimated glomerular filtration rate (eGFR), derived from the creatinine assay of the GEM^®^ Premier™ ChemSTAT, with our current laboratory analytical solution. The impact of ChemSTAT implementation on the flow of patients in the G. Montpied University Hospital (Clermont-Ferrand, France) ED was also evaluated for its potential future use.

## 2. Materials and Methods

### 2.1. Study Design

A prospective monocentric observational study was performed in the ED of the University Hospital of Clermont-Ferrand, France. The GEM^®^ Premier™ ChemSTAT (Werfen, Barcelona, Spain) was installed for 3 weeks in a dedicated study room. More than 200 remnant WB samples from the ED patients requiring the chemistry panel were evaluated on the GEM^®^ Premier™ ChemSTAT analyzer. As comparative methods, the plasma aliquots from the same samples were assayed on the Atellica^®^ CH (Siemens Healthineers, Erlangen, Germany) in the core lab. As the biochemistry residents trained by the Werfen staff carried out the analyses on ChemSTAT, the results were not reported to the referring clinician. Only the results of the core lab were used for clinical decisions.

### 2.2. Data Collection

The following data were collected:

- Patient demographic data: age and sex;
- The reason for ED admission;
- Times related to blood collection: time of collection, time of availability of ChemStat, and core lab results;
- Data relevant to imaging exams: type, use of CM, the time between the availability of serum creatinine results and the onset of imaging, need for analyses other than creatininemia (β-HCG) for imaging decision;
- Final diagnosis;
- Total ED time for each patient;
- Patient outcomes;
- Biological assay results on the GEM^®^ Premier™ ChemSTAT and the Atellica^®^ CH for Na^+^, K^+^, Cl^−^, total carbon dioxide (tCO_2_), urea, creatinine, glucose, and eGFR estimation.

### 2.3. Description of the GEM^®^ Premier™ ChemSTAT and the Atellica^®^ CH Measurement Methodology

The GEM^®^ Premier™ ChemSTAT is a POC whole-blood analyzer system for use at the point-of-care delivery (near-patient test) in a clinical setting and in a central laboratory. The instrument provides quantitative measurements of Na^+^, K^+^, Cl^−^, ionized calcium, glucose, lactate, hematocrit, creatinine, urea, total carbon dioxide (tCO_2_), pH, and partial pressure of carbon dioxide (pCO_2_) from arterial and venous samples. All tests are included in a single, self-contained, and multi-use disposable cartridge. Several derived analytes are available: anion gap, bicarbonate, urea/creatinine ratio, base excess, calculated total hemoglobin, ionized calcium normalized to a pH of 7.4, osmolality, and eGFR, providing results for either IDMS-traceable versions of the Modification of Diet in Renal Disease (MDRD) Study equation or the Chronic Kidney Disease Epidemiology Collaboration (CKD-EPI) equation.

These parameters, measured and calculated, aid in the diagnosis of a patient’s acid/base status, electrolyte, and metabolite balance. The system uses patented intelligent quality management (iQM) technology for continuous and active real-time monitoring of the analytical process, with automatic error detection, correction, and documentation of all corrective actions, which makes it simple to use in EDs and critical care settings by non-laboratorians [3]. The GEM^®^ Premier™ ChemSTAT system uses potentiometric sensors to measure electrolytes, urea, tCO_2_, pH, and pCO_2_. It uses amperometric sensors to measure glucose, lactate, and creatinine concentrations. Our reference technique is the Atellica^®^ CH analyzer (Siemens Healthineers). It is an automatic clinical chemistry analyzer designed to perform in vitro diagnostic tests on plasma samples. Regarding the parameters compared in our study, the Atellica^®^ CH uses indirect potentiometry for the measurement of electrolytes and different enzymatic spectrophotometric methods to measure tCO_2_, glucose, urea, and creatinine.

### 2.4. Study of the Clinical Concordance

In order to evaluate the clinical concordance with the decision cut-offs of the French Society of Radiology (SFR) for the risk of CIN, the Comparisons for Chronic Kidney Disease Epidemiology Collaboration (CKD-EPI) clearances between the GEM^®^ Premier™ ChemSTAT and the Atellica^®^ CH values were performed. The eGFR values were calculated using the Chronic Kidney Disease Epidemiology Collaboration (CKD-EPI) race-free equation (ethnicity data were not collected). The impact on clinical decisions based on 30 mL/min/1.73 m^2^ and 45 mL/min/1.73 m^2^ cut-offs was studied using the method of Snaith et al. [6].

### 2.5. Impact Analysis of POC Creatininemia in the ED

The availability of the GEM^®^ Premier™ ChemSTAT and the central laboratory biological results were compared, especially for patients who required medical imaging in the ED. The difference in the delay compared to the beginning of imaging was evaluated.

### 2.6. Statistical Analysis

Continuous data were expressed as medians, and interquartile ranges expressed continuous data. Normality was assessed using the Shapiro–Wilk test. The median time from the patients’ admissions into the ED to blood sampling was calculated. The comparison between the delays of the results with the GEM^®^ Premier™ ChemSTAT and the central laboratory was conducted using the Wilcoxon paired test. Relationships between continuous data were analyzed using correlation coefficients (Pearson or Spearman, depending on the statistical distribution; noted r). The agreements between the GEM^®^ Premier™ ChemSTAT WB and the Atellica^®^ CH plasma samples were assessed for each analyte using the Lin concordance correlation coefficient.

The results were interpreted in relation to the recommendations in the literature: <0.4: no agreement; 0.4–7: poor agreement; >0.7: moderate to strong agreement for Lin’s coefficients [7]; as well as, <0.1: negligible; 0.1–0.4: weak; 0.4–0.7: moderate; 0.7–0.9: strong; and >0.9: very strong, for correlation coefficients [8]. Bland–Altman plots were also plotted. The mean bias and 95% limits of agreement were calculated. In addition, for the creatinine comparison, a linear regression was performed to establish a correspondence formula between the creatinine Atellica^®^ CH and the creatinine GEM^®^ Premier™ ChemSTAT. The agreement between the methods was also examined for the creatinine categorized according to the renal risk cut-offs for CIN, established by the French Society of Radiology. More precisely, for the eGFR evaluation, the clinical concordance of WB POC results (eGFR cut-off = 30 mL/min/1.73 m^2^ and eGFR cut-off = 45 mL/min/1.73 m^2^) vs the plasma central laboratory results, was assessed. A two-tailed P-value of less than 0.05 was considered statistically significant. Statistical analyses were performed using Stata software, version 15.0 (StataCorp, College Station, TX, USA). An analysis of the method comparisons and a graphical presentation of study results were carried out using Plever Viscali software, version 6.2.

## 3. Results

### 3.1. Characteristic of the Population

Samples from 217 ED critical care patients were analyzed. The median patient age was 64 years (IQR 44–80), consisting of 94 women (43.3%) and 123 men (56.7%). General categories of admission reasons leading to an ED visit included 193 (88.9%) medical causes, 16 (7.4%) related to psychiatric pathology, and 8 (3.7%) patients admitted for surgical problems. The final diagnoses following emergency management were medical for 186 (85.7%) patients, psychiatric in 20 (9.2%) cases, and surgical for 11 (5.1%) patients. The patient’s outcome after an ED visit was as follows: hospitalization for 110 (50.9%) patients, discharge at home for 105 (48.2%) patients, and death in two (0.9%) cases.

### 3.2. Method Comparison Study

The number of samples, the sample range, the regression slope, the Pearson correlation coefficient (r), with an estimated bias, and 95% limits of agreement (Bland–Altman) were calculated for each analyte and are summarized in Table 1. The WB results from the GEM^®^ Premier™ ChemSTAT correlated well with those from the Atellica^®^ CH (Siemens Healthineers), and Table 1 summarizes the regression results. Analyte biases were estimated at each medical decision level per analyte and were all within manufacturers’ claims except for:

- tCO_2_, the bias between the comparative methods of Siemens and Roche Cobas (ChemSTAT predicate), which may be attributed to the observed low-slope and negative bias;
- Sodium and chlorine, the known bias between the comparative methods of Siemens and ChemSTAT, which is due to the difference between direct and indirect ion selective electrodes (ISE).

**Table 1:** Summary of method comparison between the GEM^®^ Premier™ ChemSTAT WB and the Atellica^®^ CH plasma samples.

Analyte	n	Min	Max	r	Slope	Mean Bias (SD)	95% Limits of Agreement
**Creatinine (µmol/L)**	217	39.4	422	0.983	1.197	19.214 (12.344)	−4.979–43.407
**Urea (mmol/L)**	206	1.7	37.54	0.982	1.237	1.587 (1.286)	−0.934–7.109
**Na^+^ (mmol/L)**	217	118	145	0.799	1.051	−2.023 (2.276)	−6.484–2.438
**K^+^(mmol/L)**	211	2.4	7.6	0.906	1.161	0.191 (0.273)	−0.344–0.725
**Cl^−^ (mmol/L)**	171	80	114	0.888	1.232	1.129 (2.38)	−3.537–5.794
**Glucose (mmol/L)**	205	3.1	52.56	0.971	0.899	−0.027 (1.140)	−2.261–2.207
**tCO_2_ (mmol/L)**	216	14.1	38.4	0.856	0.919	−2.984 (1.561)	−6.043–0.075

### 3.3. Focus on Method Comparison Study for Creatinine

**Figure 1:**
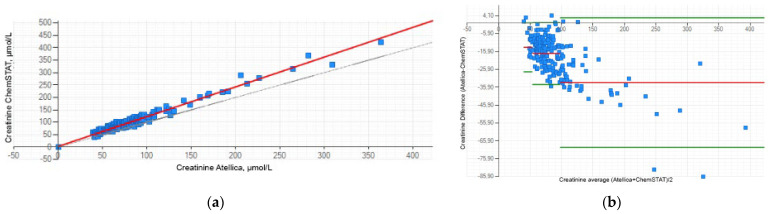
Correlation between the WB samples analyzed on the GEM^®^ Premier™ ChemSTAT and the plasma samples analyzed on the Atellica^®^ CH for creatinine: (**a**) linear regression; (**b**) Bland–Altman difference plots. The red line represents the absolute mean difference (µmol/L). The green lines represent the limit of agreement (±1.96 SD of the differences).

The relationship between the two methods can be expressed as follows: creatinine Atellica^®^ CH = 1.197 × creatinine GEM^®^ Premier™ ChemSTAT + 3.007 (Figure 1a). Lin’s concordance correlation coefficient was calculated at 0.912, which indicated a strong concordance between the two methods. The Bland–Altman plot presented a mean bias of ±1.96 SD between the Atellica^®^ CH and the GEM^®^ Premier™ ChemSTAT for three creatinine ranges (Figure 1b). Despite the positive bias, this graphic analysis revealed good agreement between the two methods. The mean difference for the low creatinine (40–53 µmol/L) sample range is −13.591 µmol/L; for the normal creatinine (54–97 µmol/L) sample range, the mean difference is −17.051 µmol/L; for the high creatinine (98–422 µmol/L) sample range, the mean difference is −33.329 µmol/L. The biases estimated are all within ±1.96 SD limits of agreement, except for some creatinine values, for which their differences do not influence the decision to administer CM.

### 3.4. Clinical Concordance Study: eGFR Evaluation

**Figure 2:**
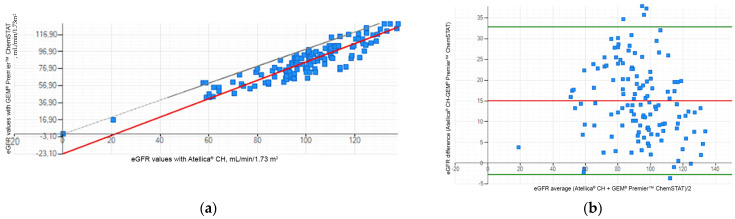
Correlation between the Atellica^®^ CH and the GEM^®^ Premier™ ChemSTAT creatinine methods for the eGFR values: (**a**) linear regression; (**b**) Bland–Altman plot.

The correlation between the Atellica^®^ CH and the GEM^®^ Premier™ ChemSTAT creatinine methods for the eGFR values (n = 128) was: r = 0.904; slope = 1.080; and intercept = −23.097 mL/min/1.73 m^2^ (Figure 2a). Lin’s coefficient of concordance between the two methods was calculated at 0.708, indicating a moderate to strong positive correlation. Additionally, a Bland–Altman plot illustrated lower values for WB eGFR of the GEM^®^ Premier™ ChemSTAT, with a mean difference between the two methods of 15.051 mL/min/1.73 m^2^ (Figure 2b). These results are a consequence of the systematic overestimation of WB creatinine compared to plasma samples, and due to the inverse relationship between creatinine and eGFR values.

**Figure 3:**
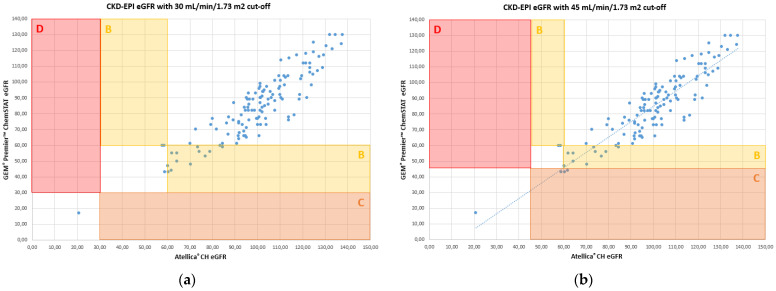
Error grid analysis of concordance between eGFR risk stratification using the GEM^®^ Premier™ ChemSTAT vs Atellica^®^ CH creatinine methods: (**a**) comparison by a 30 mL/min/1.73 m^2^ threshold; (**b**) comparison by a 45 mL/min/1.73 m^2^ threshold.

The results obtained for the creatinine assay allow for making identical medical decisions compared to the renal-risk cut-offs for CIN, which were established by the French Society of Radiology (SFR). An error grid analysis [6] for eGFR identifies the impact of the differences in results between WB and calculated plasma eGFR. The results are classified into four zones, as described by Snaith [6], and presented according to two SFR risk stratification cut-offs. Figure 3 illustrates the error grid analysis for CKD-EPI.

The results of the concordance study summarized in Table 1 indicate that WB creatinine on the GEM^®^ Premier™ ChemSTAT aligned well with the core lab for assigning a patient to a risk category. There is potential over-prophylaxis with POC creatinine for 2.3% of patients only for the cut-off of 45 mL/min/1.73 m^2^ (Zone C, Figure 3b et Table 2). No patient is misclassified for high risk of CIN (Zone D, Figure 3 et Table 2).

### 3.5. Study of the Organizational Impact of the POC Creatinine Implementation in the ED

Data on the prescription of medical imaging examinations for the patients admitted to ED are described in Table 3.

The median time from patients’ admissions into the ED to blood sampling was 15 min (min: 0; max: 207; EI: 10–21). A statistically significant difference was found between the delays of the results with the GEM^®^ Premier™ ChemSTAT versus the central laboratory (*p* < 0.001) (Table 4).

The improvement of the turnaround time for creatinine results compared to the timing of the imaging performed was noted. The availability of creatinine results for patients who required CT was faster with ChemStat. The mean difference was 45.2 min (SD 75.6). The median difference was 38 min (EI: 19–66).

However, it must be considered that POC creatinine is not always sufficient to allow for X-ray imaging. In our study, the availability of the result of the β-HCG analysis by the core lab was necessary in 9 cases (6.29%) to authorize the CT scan.

## 4. Discussion

Contrast-induced nephropathy (CIN) or contrast-induced acute injury (CI-AKI) remains significant in both ambulatory and hospitalized patients. CIN is defined as a creatinine level increase of ≥0.3 mg/dL (26.5 μmol/L) above the baseline value within 48 h of contrast media exposure or an increase of at least 1.5 times the baseline value within 7 days [11,12,13] in the absence of alternative etiologies [13,14]. Therefore, it is important to prevent it in ED patients. Indeed, this is difficult in the case of home discharge monitoring of renal function for 7 days after CM administration. As demonstrated by our study, medical imaging is very frequent in emergency rooms containing aging populations with risk factors. Pre-existing compromised renal function is a sufficient and almost necessary condition for the development of kidney aggression by CM [15,16,17]. The rapid availability of serum creatinine results with eGFR calculation was an integral part of CIN prevention in the ED. It helped to organize peri-procedural management of patients (hydration, pharmacological agents) or even alternative imaging modalities, leading to shorter hospital stays and better outcomes [13]. CM administrations in vital emergencies were performed without knowing renal profiles, but according to the results, the sample for the creatinine assay must be carried out with validated measures enterprises posteriori [16].

Previous studies have confirmed the analytical performances of the GEM^®^ Premier™ ChemSTAT [3,4,5,11]. The effectiveness of the POC solution in improving the triage of patients in the ED has been demonstrated [18]. POC creatinine can play the role of CIN prevention in different urgent clinical situations, in accordance with several studies [6,19].

As highlighted by several authors [3,4,20], the differences in methods and blood matrices lead to a bias between the POC and core lab analyzers. Thus, before adopting a new POC analyzer, it is important to evaluate its analytical performance against the existing laboratory method [4]. A comparison of the GEM^®^ Premier™ ChemSTAT with the Atellica^®^ CH was not available in the literature. This study revealed that WB results from the GEM^®^ Premier™ ChemSTAT correlated well with the plasma samples on the Atellica^®^ CH across the tested sample ranges. We have been particularly interested in the comparability of creatinine assays. Current studies confirmed our comparisons with the previous Siemens method of the central laboratory [5]: there is a slight overestimation of creatininemia with the ChemSTAT method, accentuated by the sample matrix effect, as already mentioned [4]. Therefore, it is important to study the clinical concordance of POC creatinine eGFR results using the central method. The concordance between the eGFR risk stratification POC ChemSTAT creatinine and the laboratory creatinine assay was evaluated by applying the error grid analysis first proposed by Snaith et al. [6]; the method adopted by authors studying the same subject [3,21,22]. Unlike other studies, we evaluated two eGFR thresholds for the stratification of CIN risk in order to meet current recommendations of 30 mL/min/1.73 m^2^ and 45 mL/min/1.73 m^2^ [9,10]. The analysis indicates the safety of creatinine assays with the GEM^®^ Premier™ ChemSTAT for eGFR risk stratification. There were a small number of misclassified samples for the two cut-offs, with no impact on CM administration decisions. If the threshold of 45 mL/min/1.73 m^2^ must be considered, 2.3% of patients risk a potential delay because of an unnecessary prophylaxis. In addition, this study demonstrates that no patient presents an increased risk of CIN with the GEM^®^ Premier™ ChemSTAT creatinine assay.

In our study, the POC method decreased turnaround times for biological results by approximately 50 min compared to the central laboratory. This is the first study that analyzes the impact of the GEM^®^ Premier™ ChemSTAT in an ED, but this result is similar to other POC analyzers [22,23]. We were particularly interested in the impact of the availability of WB creatinine on the risk stratification of patients before CT and with CM administration. In our conditions, the expectation results for eGFR were reduced by an average of 45 min, allowing for the reduction in waiting times to schedule CT and to plan preventive measures when necessary. In a previous study, this time was estimated to be 20 min [24], and the authors deduced the possibility of one to three additional scanners per day. This result varies depending on the laboratory organization and clinical practices. Certainly, this potential optimization of the organization of medical imaging warrants an evaluation under our conditions. In addition, the availability of POC creatinine is important to reduce the incidence of canceled CT scans [22].

The main limitation of our study lies in the small number of patients with severe (plasma eGFR < 30 mL/min/1.73 m^2^) renal insufficiency, but this population is representative of the ED patients.

## 5. Conclusions

The GEM^®^ Premier™ ChemSTAT can be safely used to stratify the risk of CIN in patients admitted to the emergency department who require medical imaging with a contrast media injection. The availability of WB creatinine can improve the flow of patients in EDs by optimizing access to medical imaging. Further studies are needed to validate the cost-effectiveness of using POC creatinine in emergency care settings.

## Figures and Tables

**Table 2:** Clinical concordance and risk stratification between the WB GEM^®^ Premier™ ChemSTAT and the plasma Atellica^®^ CH eGFR.

	Number of Patients (%)	
Zone ^a^	30 mL/min/1.73 m^2 b^	45 mL/min/1.73 m^2 c^
**Zone A**: Correct risk classification—appropriate management.	114 (89.1%)	114 (89.1%)
**Zone B**: Incorrectly classified, but no implication for clinical management.	14 (10.9%)	11 (8.6%)
**Zone C**: Incorrect classification, potential for unnecessary prophylaxis, or withholding of contrast.	0 (0%)	3 (2.3%)
**Zone D**: Incorrect classification and potential for increased risk of CIN owing to insufficient prophylaxis.	0 (0%)	0 (0%)

^a^ Performance zones for risk categorization based on the CKD-EPI eGFR calculations, as defined by Snaith et al. [6]. ^b^ Renal-risk cut-off of eGFR < 30 mL/min/1.73 m^2^. Preventive measures are recommended before intravenous and intra-arterial CM administration, with second-pass renal exposure [9,10]. ^c^ Renal-risk cut-off of eGFR < 45 mL/min/1.73 m^2^. Preventive measures are recommended before intra-arterial CM administration, with first-pass renal exposure, and for intensive care unit patients [9,10].

**Table 3:** Summary of imaging techniques in the ED.

Imaging Exams	N Patients (%)	%
No prescribed	74	34.10
Radiography	46	21.20
Computed Tomography:	97	44.70
- CM administration	66	68.04
- without CM	31	31.96

**Table 4:** Summary of the turnaround time data for POC analyzer and core lab.

Interval Between Blood Sampling and Result Available for Viewing	Median, in Minutes (IQR)	Mean, in Minutes (SD)
GEM^®^ Premier™ ChemSTAT	26 (17–37)	36.2 (36.7)
Central laboratory	67 (53–94)	86.1 (60.0)

## Data Availability

Data supporting results can be obtained from the authors.
